# A psycholinguistic study of intergroup bias and its cultural propagation

**DOI:** 10.1038/s41598-024-58905-y

**Published:** 2024-04-14

**Authors:** Daniel Schmidtke, Victor Kuperman

**Affiliations:** Department of Linguistics and Languages, McMaster University, Hamilton, L8S 4L6 Canada

**Keywords:** Human behaviour, Language

## Abstract

Intergroup bias is the tendency for people to inflate positive regard for their in-group and derogate the out-group. Across two online experiments (N = 922) this study revisits the methodological premises of research on language as a window into intergroup bias. Experiment 1 examined (i) whether the valence (positivity) of language production differs when communicating about an in- vs. out-group, and (ii) whether the extent of this bias is influenced by the positivity of input descriptors that were initially presented to participants as examples of how an in-group or out-group characterize themselves. Experiment 2 used the linear diffusion chain method to examine how biases are transmitted through cultural generations. Valence of verbal descriptions were quantified using ratings obtained from a large-scale psycholinguistic database. The findings from Experiment 1 indicated a bias towards employing positive language in describing the in-group (exhibiting in-group favoritism), particularly in cases where the input descriptors were negative. However, there was weak evidence for increased negativity aimed at the out-group (i.e., out-group derogation). The findings from Experiment 2 demonstrated that in-group positivity bias propagated across cultural generations at a higher rate than out-group derogation. The results shed light on the formation and cultural transmission of intergroup bias.

## Introduction

Intergroup bias is a well-studied and arguably universal human tendency to categorize social groups into *us* and *them*, with a preference for *us* (the in-group) over *them* (the out-group)^1–3^. While intergroup bias is a force that can lead to in-group favoritism—the enhancement of positive regard toward in-group but not out-group members^4^—it may also engender out-group derogation, i.e., the active extension of negativity toward the out-group^5^. The popularity of intergroup bias as a topic is not incidental. Forms of intergroup bias include prejudice and stereotyping^6,7^ based on disability^8^, race^9^, religion^10^, gender identity^11^, and sexual orientation^12^, biases that are often linked to discrimination^13–15^, hostility^16–18^, and violence^19–22^. The present study focuses on how intergroup bias is encoded in language usage. Using tools of lexical semantics, combined with experimental methods from cultural evolution, we propose a new quantitative method of studying the formation and cultural propagation of intergroup bias.

A well-established finding in the study of intergroup bias is that attitudes about the in-group tend to be more positive than those about the out-group^2,5, 23–25^. People tend to trust in-group members more than out-group members^26^, evaluate in-group members more positively than out-group members^27^, and are more likely to cooperate with in-group members than with out-group members^28^. Moreover, multiple streams of evidence converge on the idea that intergroup bias is asymmetric: intergroup discrimination is best characterized as love directed towards one’s in-group rather than strong and active hostility towards an out-group (for reviews see^29,30^). For example, people are more likely to provide help to their own race (i.e., in-group) rather than another (i.e., out-group)^31^, and in-group members are more likely to favour their own group in the allocation of rewards and penalties over out-group members^32^.

Measures of psychological valence (the negative-positive scale) have long been a part of the experimental toolkit examining explicit and implicit bias, e.g. priming and self-report measures^2,33^. In studies where the valence of linguistic stimuli was the critical experimental manipulation, a common approach has been for verbal stimuli to be selected based on researcher intuition and then grouped into categories to form a discrete dichotomous variable with 2 levels: ‘positive words’ vs. ‘negative words’, but see^34,35^. A demonstration of this experimental set-up can be seen in a priming study focused on implicit attitudes^36^. In this study, individuals exhibited faster response times when categorizing a target adjective as either ‘good’ or ‘bad’ if the valence of the target adjective and preceding prime word both aligned. For example, relative to controls, response time facilitation was found when people correctly categorized positive target words, e.g., *delightful*, as ‘good’ when preceded by positive primes, e.g., *friend*, and when classifying negative target words, e.g., *awful*, as ‘bad’ when preceded by negative primes, e.g., *landlord*. Additional variations of this priming technique with similar experimental designs have been used to examine general mechanisms of intergroup bias^27^ and the specific ways in which intergroup bias manifests in society, such as in racial attitudes^37^ and ageism^38^. In addition to using intuitions about valence for stimulus selection, researchers of intergroup bias have also used intuitions about the valence of *language usage* as an outcome variable. Most relevant studies adopting this approach have relied on manual annotation of verbal productions as positive, neutral, or negative (with a slight variation in the coding schemas, and the number and quality of categories for annotation), see e.g., two independent and study-blind coders providing annotations^39,40^, or two judges rating ethnophaulisms on a 1-7 negative to positive scale^41^.

Another influential body of research that relies on the measurement of verbal valence is the line of investigation devoted to Linguistic Intergroup Bias (LIB)^42^. LIB is the tendency to describe positive behaviors of the in-group and negative behaviors of the out-group using more abstract lexical categories, but to describe negative behaviors of fellow in-group members and positive behaviors of the out-group using concrete language (for reviews see^43,44^). Much of the prior experimental research on the LIB has relied upon measurements of valence derived from language usage that is freely produced by participants in intergroup conditions^42,45, 46^. However, similar to the priming studies described above, the valence of linguistic descriptions is often coded by researchers themselves^47,48^. In addition, this researcher-based coding has been used to study LIB in naturally occurring groups outside of the laboratory, such as in linguistic studies of free-form survey responses about local political, sports, and religious groups^49^, and in text analyses of newspaper articles describing out-group nations^50^.

An alternative method for assessing intergroup bias involves utilizing the Linguistic Inquiry and Word Count (LIWC) database^51^. LIWC is a set of tightly curated, manually selected words and phrases that are associated with specific emotions and mental states. Texts produced by participants are evaluated on how many of the words from a certain list they contain: A higher percentage of words from, say, the list associated with positive emotions signify a more positive outlook of the text. One way this corpus has been used in intergroup bias research is to examine emotional content of language used in an online bias reduction program aimed at reducing prejudice between Muslims and Christians^52^.

This paper presents an additional approach to quantifying valence in research on bias, useful both for stimulus selection and for reliable evaluation of participants’ verbal productions. This approach relies on the use of the large database of linguistic norms of psychological valence, and thus avoids some methodological pitfalls of other approaches. Below we present two experiments on the group bias and demonstrate the utility of using existing databases of psycholinguistic information.

### The present study

The present study describes two simple experiments in which we focused on testing the hypothesis that attitudes about the in-group tend to be conveyed with more positive language compared to attitudes about the out-group^2^. The experiments are motivated by the observation that precise measurements of lexical valence are of paramount importance yet are rarely employed in intergroup bias research. We addressed this issue by harnessing Warriner et al.’s^53^ large-scale lexical database containing fine-grained norms of valence for tens of thousands of words. Large-scale corpora containing norms of a wide array of word properties are abundant, available in multiple languages, and are now conveniently accessible through comprehensive online ‘metabases’^54^. Lexical norms from databases are routinely accessed by researchers to obtain stimuli for experimental use. For example, a common practice in psycholinguistic research design is to generate experimental stimuli in a ‘bottom-up’ fashion, such that experimental stimuli are randomly sampled from the available databases that represent a range of values for the desired lexical variable(s)^55–57^.

Our approach of drawing the affective ratings of words from an independent database, we believe, is imperative for the study of intergroup bias. We argue that there are at least two reasons for adopting this approach. The first is that Warriner et al.’s (2014)^53^ database of affective ratings for words was not motivated by the goal of testing a predefined hypothesis or a theory regarding intergroup bias. This characteristic enhances the validity of our study, as the database was compiled independently, without being influenced by researcher biases or preconceptions related to inter-group dynamics. Consequently, the database provides a valuable resource for investigating inter-group bias in a manner untethered from theory. This advantage is apparent in how the database has been employed to investigate various phenomena, including word recognition in the visual and auditory modalities^58^, anxiety and attentional bias in children with specific learning disorders^59^, word learning^60^, and comparisons of language usage on social media across nations^61^. The second way in which the present database is useful lies in the scope of data that it affords the researcher. Warriner et al.’s database contains average valence ratings for roughly 14,000 English words, which represents a significant proportion of the existing English word stock. This is a major advantage over other vocabulary-based approaches, including the use of the Linguistic Inquiry and Word Count (LIWC) database^51^. For instance, the LIWC database covers the positive and negative semantic dimensions of a few hundred words: The presence and percentage of these words in texts is arguably sufficient to indicate the emotional coloring of texts. Yet the number of those manually curated words is small (roughly 600 words in LIWC for positive valence vs. 14,000 words in Warriner et al. with valence ratings) and they are not able to be differentiated based on degree of positive emotion (e.g., *love* and *readiness* are both found in the positive emotion list and thus are equally impactful in the analysis using LIWC, yet their valence ratings in Warriner et al. are 8.00 and 6.24 respectively, a difference of 22% on the 1–9 valence scale). In the present study, we make use of the availability of valence ratings for thousands of words in Warriner et al.’s database both to generate stimuli and to gauge the positivity of language output. Specifically, in the present set of experiments on intergroup bias we use the database (i) to maintain a precise and controlled restriction of the emotional positivity of the generated lexical stimuli that participants are shown as *input*, and (ii) to quantify the valence of words that are freely produced by participants as behavioral *output*.

Experiment 1 of the present paper aimed to establish whether corpus-based estimates of lexical valence can be used as a reliable tool for detecting the positivity bias in intergroup scenarios. In the experiment, participants were informed that they belong to either an in-group or out-group and were provided with a set of “seed” words. Participants in the “in-group” condition were told that the seed words describe their in-group and, based on these descriptions, were instructed to provide three more words to describe their own group. Participants in the “out-group” condition were told that the seed words describe an out-group and were asked to provide three additional words to describe that group. The lexical valence estimates of the words produced by the participants served as the outcome variable. This enabled us to gauge the effect of intergroup bias on written verbal productions. We also systematically manipulated the valence of the randomly sampled seed words so that they ranged from highly negative to highly positive. This allowed us to tightly control the positivity of the “ground truth”, i.e., the input information that was provided about the group that is being evaluated, defined as the average word valence rating in Warriner et al.’s database.

We examined the difference in positivity between the input seed and verbal descriptions with the aim of quantifying how strongly ground truth is distorted by intergroup bias. We tested a series of hypotheses in this regard. First, it is possible that the valence of the evaluations of the in-group will be positively inflated relative to the ground truth while the valence of the descriptions about the out-group match the seed, i.e., in-group favoritism^2^. Another hypothetical scenario is that descriptions about the out-group are more negative compared to the ground truth while the valence of the descriptions remain stable, i.e., out-group derogation. It is also possible to observe that both in-group and out-group evaluations diverge from the seed valence, i.e., in-group favoritism *and* out-group derogation. Finally, it is possible that these patterns depend on the level of the seed valence. For instance, in-group favoritism may only occur when the ground truth is especially negative.

Building upon Experiment 1, the goal of Experiment 2 was to examine the transmission and propagation of intergroup bias across cultural “generations”. Borrowing from cultural and language evolution research, we utilized the *linear diffusion chain* paradigm^62–65^, an experimental paradigm which exposes a group of participants to an artificial language task with a given input, collects behavioral responses based on this input, and then feeds these responses as input to the next generation of participants facing the same task. Also known as the *iterated learning* paradigm, studies adopting this method have shown that from an initial random state, structural properties of language increase and become easier to learn as they are passed through each successive generation of participants. Relevant to this study, Martin et al.^64^ used the iterated learning paradigm to examine how stereotypes are formed, and found that information about novel ‘alien’ targets became more simplistic, structured, and more learnable as information about aliens and their attributes were transmitted through generations of participants. The authors argue that the gradual changes toward simpler and more easily transmissible (i.e., learnable) information about social targets and their features provides clues to the cognitive mechanisms by which stereotypes are formed in the culture.

**Figure 1:**
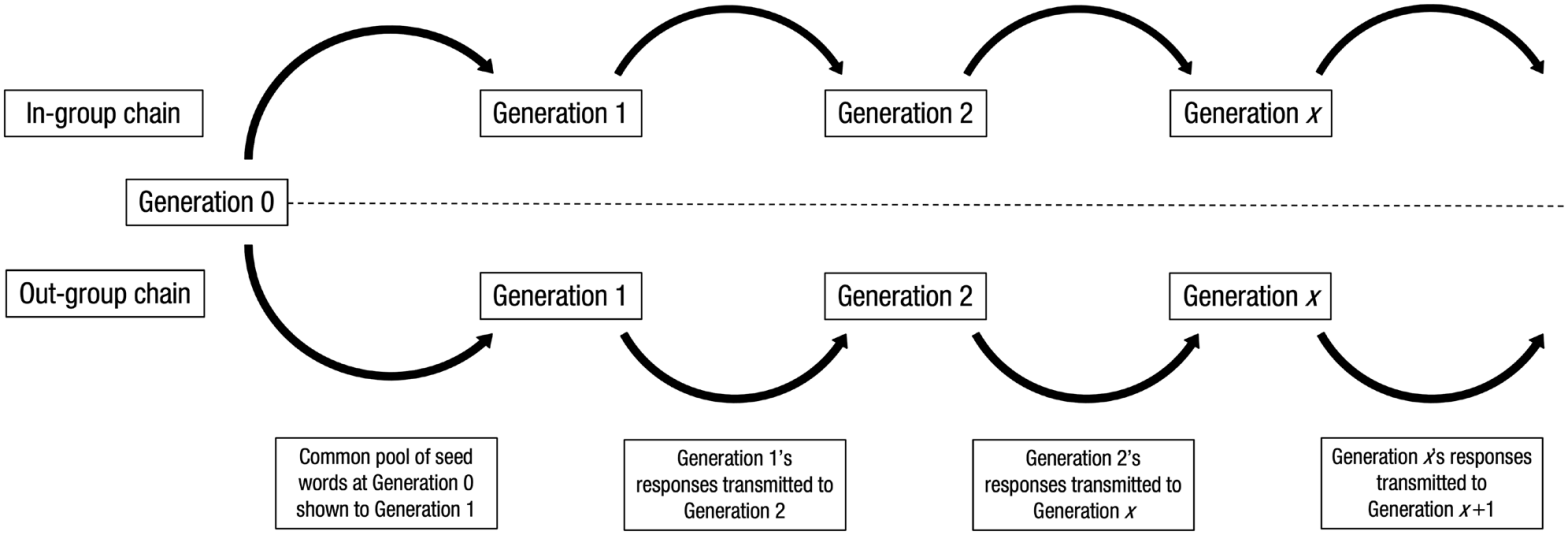
Graphic illustrating the linear diffusion chain method adapted from Martin et al.^64^ and applied to the social-transmission of intergroup bias. The in-group and out-group chain each began with the same set of three seed words (Generation 0). The first group of participants in each chain (Generation 1) were shown the seed words and were told that they described a target group: either their own group (if they belonged to the in-group chain condition) or an out-group (if they belonged to the out-group chain condition). Participants were then asked to provide three additional words to describe either their own group (if in the in-group chain) or the out-group (if in the out-group chain). For each chain, a randomly selected subset of 12 words provided by each group was used as the input for the next group of participants in that same chain (i.e., Generation 2). This procedure was repeated four times per chain to produce transmission chains of five generations.

While Martin et al.^64^ focused on the transmission of information structure during stereotype formation, the aim of Experiment 2 was to use the linear diffusion chain method to address a previously unexamined aspect of cumulative cultural evolution. Namely, we investigated whether affective information that is encoded in language usage is detected and altered as it is transmitted from person to person. We applied the linear diffusion chain method to the task described for Experiment 1 and investigated the transmission of evaluations towards in-groups vs. out-groups based on an initial set of seed words (Fig. 1). Using this paradigm, we explored whether intergroup bias effects in valence amplify, diminish, or remain stable over cultural generations, and whether affective biases in descriptions of in- vs. out-groups change at the same rate across generations.

## Results

### Experiment 1: Lexical valence as a diagnostic of intergroup bias

Three patterns in the data are of interest: (i) how different in-group and out-group descriptions are from one another, (ii) how closely the written descriptions of the in-group and out-group approximates the input (seed) valence, and (iii) whether the magnitude of the inflation or deflation of the valence in-group and out-group responses, if found, is related to the seed valence.

**Figure 2:**
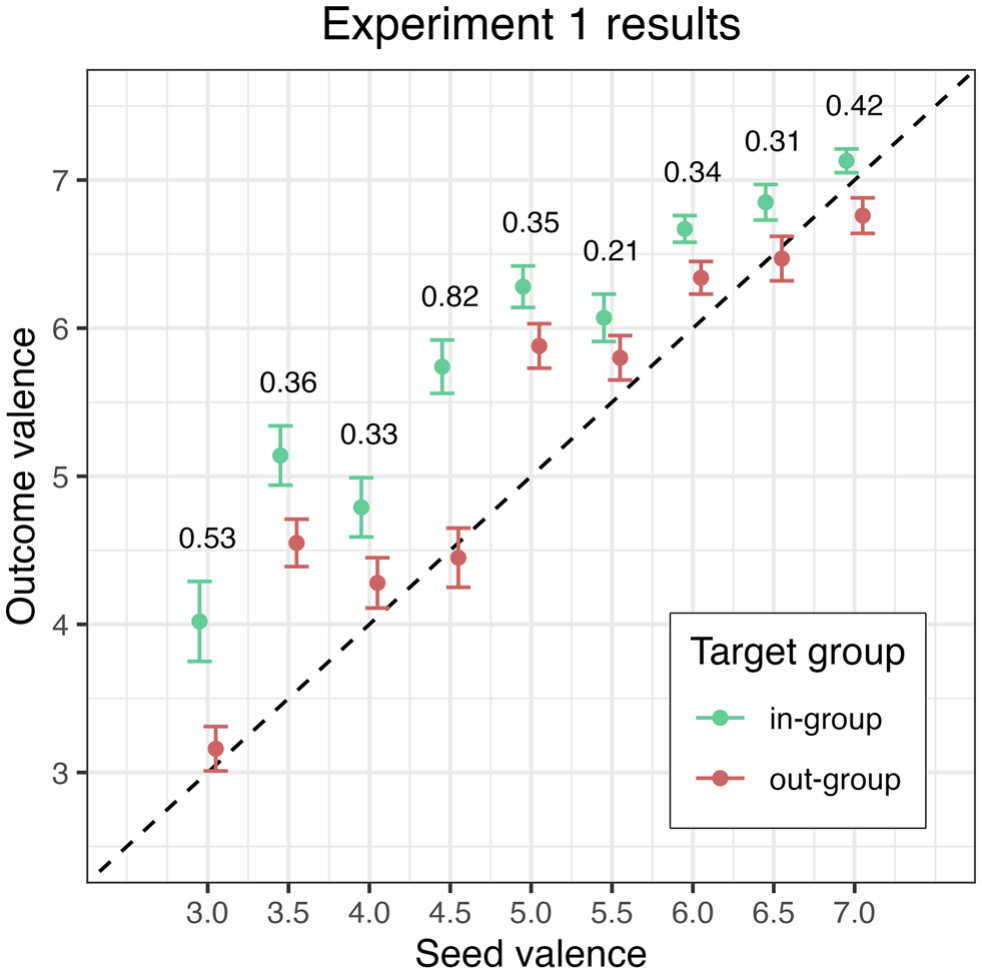
Valence of responses in the completion task in Experiment 1 as a function of seed valence and target group. Error bars stand for ± 1 SEM. Cohen’s *d* effect sizes are reported for the contrast between in- and out-groups at each seed valence level.

#### How different are in-group and out-group evaluations?

It is evident from Fig. 2 that the valence of verbal responses was higher for the in-group condition compared to the out-group condition. A two sample *t*-test confirmed that there was a significant difference between the average valence of responses for the in-group condition (*M* = 5.92, *SD* = 1.65) and the out-group condition (*M* = 5.34, *SD* = 1.74; *t*(1301.52) = 6.18, *p* < .001; Cohen’s *d* = 0.34). Furthermore, the differences between the in- and out-group valence (i.e., the contrast between the red and the green dots in Fig. 2) reached statistical significance (*p *< .05) in five out of nine *t*-test comparisons (seed levels 3.0, 3.5, 4.5, 6.0, and 7.0, at the 5% level after correction for multiple comparisons). These results confirm the hypothesis that verbal responses towards the in-group tend to be more positive compared to when directed towards the out-group.

#### How close are the in-group and out-group descriptions to the seed valence?

The data plotted in Fig. 2 shows that evaluations about the out-group were much closer to the input valence (the dotted line) than those for the in-group. One-sample *t*-tests comparing the mean valence of responses for the out-group with seed valence (i.e., the red dots compared to the dotted line, in Fig. 2) failed to reach significance for six out of nine levels of seed valence. The contrasts that did reach statistical significance were at seed levels 3.5, 5.0, and 6.0. Concerning the series of *t*-tests for the contrasts between the mean valence of responses for the in-group and seed valence (i.e., the green dots compared to the dotted line, in Fig. 2), eight out of nine *t*-tests (after correction) showed a significant inflation of valence in the positive direction (only seed level 7.0 did not show a significant increase in response valence). Therefore, affective evaluations of the out-group tended to be closer to the “ground truth”, while those of the in-group were consistently inflated.

#### Is the extent of inflation or deflation in the valence of verbal descriptions related to seed valence?

It can be seen in Fig. 2 that, relative to the seed level valence, the inflation of positivity for the in-group responses tended to be larger when the seed valence was more negative. To take an example from each extreme of seed valence, there was a 1.02 point inflation in the average valence of in-group responses for the minimum level of seed valence (3), yet there was a 0.13 point inflation in the average valence of in-group responses for the maximum level of seed valence (7). This association between seed valence and the amplification of bias does not appear to be present for the out-group. To examine this effect further, for each group, we (i) computed the difference between the seed valence and the valence of each individual verbal response, and then (ii) correlated these difference values with seed valence. For in-group responses, the estimated correlation coefficient was *r* = − 0.26 (*p *< 0.001), which indicates that the amplification of positive regard towards the in-group was lower when the valence of the input (seed) was higher. On the other hand, this relationship was noticeably weaker for the out-group (*r* = − 0.15; *p* < 0.001). Fisher’s *r*-to-*z* conversion estimated the difference in correlation strengths to be significant (*z* = − 2.07, *p* = 0.039). We further tested this result by fitting a linear mixed-effects model to the difference scores. The fixed effects of the model included Group, Seed, and the Group × Seed interaction. The random effects of the model included by-participant intercepts (full model fitting procedures can be found in Statistical considerations, Experiment 2). The omnibus effects of the model with Type III comparisons indicated that the Group × Seed interaction was significant [*F* = 4.027(1,487.65); *p* = 0.045] (the full linear mixed-effects model can be found in Table S1 in Online Supplementary Materials). The results of these analyses therefore suggest that the inflation of the in-group was most prominent when the evaluation was based on negative input. The findings thus imply that a more negative description about one’s in-group (the seed) tends to prompt a greater urge to dissociate from this negative characterization about one’s own group behavior. Asymmetrically, the drive to dissociate is found to a smaller degree when the out-group is associated with positive emotions.

Taken together, intergroup bias was clearly present in the valence of verbal responses that were elicited in the completion task. Regardless of how positive or negative the input was, the in-group was associated with more positive evaluations than the out-group. The results support one of the possible scenarios outlined in the Introduction, one in which the difference between in- and out-group evaluations is characterized by an inflation of the positive evaluation of the in-group rather than by amplified derogation of the out-group. Finally, the positivity bias was particularly acute when the input descriptions of the in- and out-group were negative.

### Experiment 2: the cultural transmission of intergroup bias

**Figure 3:**
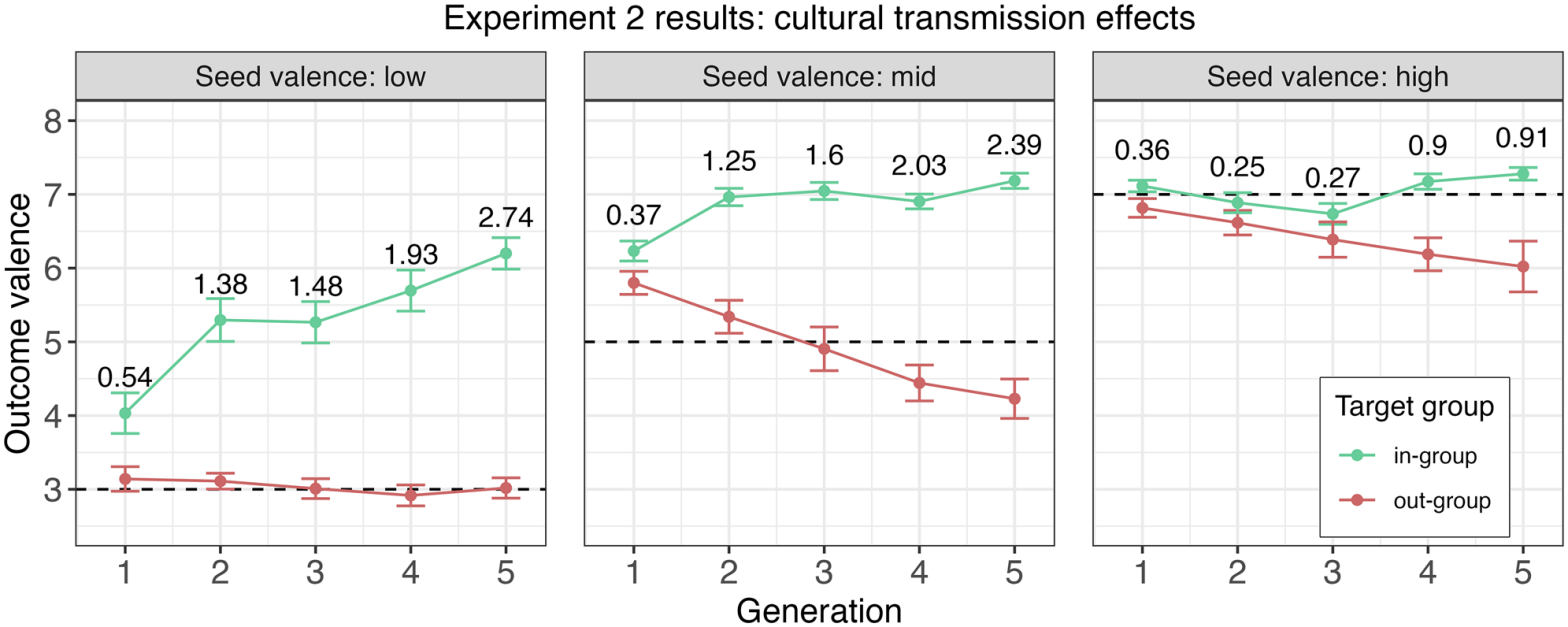
Valence of responses in a completion task in Experiment 2 as a function of group affiliation over five generations broken down by low, mid, and high-valence seed words (left, middle, and right panel). Error bars stand for ± 1 SEM. Horizontal dashed lines indicate valence of the first-generation seed words. Cohen’s *d* effect sizes are reported for the contrast between in- and out-groups at each generation.

A plot of the results from the linear diffusion chain experiment is provided in Fig. 3 and reveals several noticeable patterns. First, there was a general trend of inflation throughout the in-group chains, indicative of a gradual increase in positivity directed towards the in-group across cultural generations. The same was true for the negativity bias within the out-group chains: with the exception of the low seed valence chain, there was a steady decline in positivity directed towards the out-group at each subsequent generation. As a result, the Cohen’s *d* effect size estimates for the contrast between verbal descriptions of the in-group vs. out-group increased steadily.

**Table 1:** Omnibus effects in the analysis of valence of verbal descriptions in the cultural transmission experiment.

Effect	*F*(*df*, *df*_res_)	*p*
Intercept	224.69 (1,568.18)	< 0.001
Target group	1.60 (1,558.67)	0.21
Generation	53.55 (1,572.83)	< 0.001
Seed valence	59.43 (2,544.13)	< 0.001
Target group × Generation	33.59 (1,561.26)	< 0.001
Target group × Seed valence	0.61 (2,549.66)	0.54
Generation × Seed valence	13.08 (2,549.87)	< 0.001
Target group × Generation × Seed valence	5.6 (2,554.62)	< 0.01

We conducted statistical analyses to examine whether intergroup bias amplifies over cultural generations, and to discern whether the rate of amplification in bias directed toward the in- and out-group is contingent on the valence of the seed words. A series of linear mixed-effects regression models were fitted to valence of the written responses (see Statistical considerations). The best fitting model included a three-way interaction of seed valence, target group, and generation in predicting valence of verbal responses. Table 1 provides the omnibus effects of the final model which were calculated with Type III model comparisons. The full linear-mixed regression model, including estimated contrasts for the lower-order two-way interactions and main effects, can be found in Table S2 in Online Supplementary Materials. Here we report the results of the significant three-way interaction including (i) the estimates of the individual slopes (denoted by ${\hat{\beta }}$) for the effect of generation on in-group and out-group responses at each seed valence level (2 groups × 3 seed valence levels = 6 slopes), and (ii) a comparison of the slopes using *post-hoc* comparisons estimated from the same model. The *p*-values for the contrasts were adjusted for multiple comparisons (see Statistical considerations for full details on the statistical method).

#### Does intergroup bias amplify over cultural generations?

For verbal evaluations directed towards the in-group, the positive effect of generation was significant in the low [${\hat{\beta }}$ = 0.549; *SE* = 0.075; *p* < 0.001] and mid [${\hat{\beta }}$ = 0.203; *SE* = 0.066; *p* = 0.002] valence chains, but not in the high valence chain [${\hat{\beta }}$ = 0.038; *SE* = 0.068; *p* = 0.58]. For descriptions aimed at the out-group, the negative effect of generation was significant in the mid [${\hat{\beta }}$ = − 0.424; *SE* = 0.074; *p* < 0.001] and high valence chain [${\hat{\beta }}$ = − 0.163; *SE* = 0.077; *p* = 0.036], but not in the low valence chain [${\hat{\beta }}$ = − 0.047; *SE* = 0.076; *p* = 0.54]. These results confirm the patterns presented in Fig. 3, which exhibit a linear amplification of biases across generations, with the slowest rate of change appearing at the extremes of the valence scale. Numerically speaking, the positivity of verbal evaluations towards the in-group grew stronger across generations for the low and mid condition, but was at the ceiling for the high valence condition (Fig. 3, left and middle panels vs. right panel, green points). Conversely, the evaluations towards the out-group was at the floor in the low valence condition, but became increasingly negative across generations in the mid and high valence conditions (Fig. 3, left and middle panels vs. right panel, red points). In the next section, we examine the statistical significance of the differences in slopes.

#### Does the rate of change in the amplification of intergroup bias across cultural generations depend on seed valence?

The significant three-way interaction term (Table 1) and the tests of the slopes (see Does intergroup bias amplify over cultural generations?) point to differences in the rates of change in valence between the in-group and out-group when not constrained by floor or ceiling effects. Statistical tests of the decomposed three-way interaction in the final model confirmed the reliability of this pattern of results. The inflation in positive evaluations towards the in-group was stronger when the seed words were strongly negative rather than neutral (Fig. 3, left vs. middle panel, green points). The *post-hoc* estimated contrast between these slopes was significant [$\Delta {{\hat{\beta }}}$ = 0.346; *df* = 562; *SE* = 0.1; *p* < 0.01]. Conversely, even when there was no range restriction to dampen the amplification of negativity across generations, the increasing negative evaluation regarding the out-group was numerically stronger when the input was neutral rather than positive (Fig. 3, middle vs. right panel, red points): the difference in slopes did not reach significance at the 5% level [$\Delta {{\hat{\beta }}}$ = − 0.261; *df* = 562; *SE* = 0.11; *p* = 0.154].

In sum, this set of patterns highlights and adds nuance to the asymmetry observed in Experiment 1. The cultural transmission of bias towards enhancing an initial negative description of one’s in-group appears to be stronger than the bias towards derogating the positive traits of the out-group (Fig. 3, left panel). The results of Experiment 2 show that the rate of divergence in emotional attitudes towards the in-group vs. the out-group across cultural generations is more drastic when both groups are initially characterized using negative language than when both groups are described in positive terms.

## Discussion

This study revisited the methodological practices of the body of research dedicated to intergroup bias—the psychological preference for the in-group rather than the out-group. The current methodological re-examination of intergroup bias was motivated by the imprecise measurement of psychological valence (the negative-positive scale), an important language-based index of intergroup bias. The present study addressed this issue by harnessing fine-grained valence norms found in psycholinguistic mega-studies^53^.

First, we found an intergroup bias effect on valence in language usage, corroborating well-established findings from prior research^5,23^. Overall, written descriptions about the in-group were more positive than those about the out-group. A novel aspect of this finding was that the positivity of the written evaluations about the out-group remained very close to the “ground-truth” (measured as the valence of the seed words), while those of the in-group were systematically inflated. This finding addresses the research question about the direction of the intergroup bias effect on valence of language usage, corroborating the view that intergroup bias manifests as in-group favouritism rather than out-group derogation^24,30, 66^. Interestingly, we also found that in-group favoritism tended to be stronger when the “ground-truth” regarding the in-group was more negative. These results suggest there is an implicit tendency to dissociate one’s own group from characterizations that link one’s group with especially negative traits or behaviors. In sum, these findings demonstrate that intergroup bias is expressed verbally not as out-group derogation, but rather as in-group favoritism, especially when the ground-truth about the in-group is negative. To our knowledge, this study is one of the first to quantify this inter-group bias effect using valence ratings obtained from an independent large psycholinguistic database.

In Experiment 2, we examined the cultural transmission of attitudes and formation of stereotypes regarding in- and out-groups throughout chains of multiple generations of participants. The results of the linear diffusion chain experiment demonstrated the self-perpetuating and -enhancing nature of the attitudinal differences between the in- and out-group as they passed through groups from generation to generation. The in-group favoritism observed in Generation 1 (i.e., Experiment 1) was further amplified with every subsequent generation: the initially more negative descriptions gradually became more negative and the originally more positive descriptions became even more positive. Barring ceiling and floor effects, the rate of the cultural transmission of valence in language was virtually linear. This effect occurs despite participants in different generations being encapsulated from one another. Numerically, the trend of enhancing the negative outlook of one’s own in-group through favoritism was stronger than the bias of tempering the positive traits of the out-group. To our knowledge, this set of findings is novel and is due to the increased precision brought about with the use of psycholinguistic ratings.

In the pared-down and rudimentary verbal scenario of the present study, participants were provided with a limited number of words that described their in-group or an out-group without additional context about the features that might differentiate each group. We deliberately selected this “bare-bones” approach to stress-test the ability of the method to provide meaningful numerical estimates of intergroup bias in language. We believe that the present paper demonstrates this ability both in the original group comparison and in the linear diffusion chain scenario. It uncovers new findings and critically revisits established theories in the field. However, future research is needed to bring further nuance to the trends observed here, such as introducing richer and more ecologically valid contextual information about the in- and out-groups.

In summary, intergroup bias was clearly present in the valence of verbal responses elicited in the completion task. There was a clearer bias toward in-group favoritism rather than out-group derogation, though there was evidence that out-group derogation emerges after multiple cultural generations. The positivity bias was particularly high when in- and out-groups were described in negative terms, suggesting that when either group is badly reputed, the emotional information encoded in language is used as a cue to differentiate *us* from *them*.

## Methods

All experiments were cleared under the McMaster University Research Ethics board (protocol 2011-165) in accordance with the Canadian Tri-Council Policy Statement: Ethical Conduct for Research Involving Humans. All participants provided consent by clicking a button. Data collection took place between 27 October 2017 and 12 December 2018.

### Experiment 1

The experiment involved a task (described below) in which participants were shown an initial set of common “seed words”, and based on these words were asked to provide a written description about a fictitious “target group”, either the in- or out-group. Participants were asked to provide three additional words to describe their target group. This experiment had a factorial (2 × 9 design). The first factor is Group (2 levels: in-group and out-group) and is a between-subject factor referring to the group that the verbal descriptions are aimed at. The second between-subjects factor is the valence of the seed words. We manipulated the seed words such that they spanned 9 discrete levels of psychological valence, from very negative to very positive (operationalized below).

#### Participants

A total of 502 participants were recruited through the crowd sourcing platform Amazon Mechanical Turk (https://www.mturk.com). Participants were randomly assigned to one of the two target group conditions (*n* in-group = 245; *n* out-group = 257). Participants were also randomly assigned to the set of seed words associated with one of the nine valence levels (median *n* per condition = 27; min *n* = 21; max *n* = 35). Inclusion criteria consisted of residence in Canada or the USA and successful prior completion of at least 90% of the tasks on Amazon Mechanical Turk. Participants were rewarded with 0.30 USD for completing the task. Because of the constraints imposed by Amazon Mechanical Turk, participants were able to complete the task multiple times in multiple conditions (described below), but only their first set of responses were included in the analyses.

#### Stimulus materials

Nine sets of seed words representing specific points covering the entire range of emotional valence were created. Our selection was obtained from an existing database of valence norms of 14,000 English words^53^, where words were rated on a scale from 1 (unhappy) to 9 (happy). Since the vast majority of the words have ratings between 3 and 7, our word selection only covered this range. We selected words from nine levels of valence between 3 and 7, with a step of 0.5 points on the valence scale (e.g., 3.0, 3.5... 6.5, 7). Words with valence ratings within a very narrow band (absolute deviation $<=$ 0.1) from each level were identified (e.g., words with valence ratings between 3.4 and 3.6 represent the target level of 3.5). At each level we randomly selected three adjectives to represent the given target level of valence, e.g., *unrelenting*, *vulnerable*, *suspicious* for the target valence level of 3.5. Table 2 reports the resulting nine sets of seed words, along with their associated valence levels.

**Table 2:** Seed words for Experiment 1. Words selected at 9 levels across the valence range.

Seed valence	Stimuli
3.0	Despicable, guilty, slimy
3.5	Compulsive, hairless, imperial
4.0	Irregular, overwhelming, pricey
4.5	Experimenting, mythical, negotiable
5.0	Acute, instantaneous, meticulous
5.5	Suspicious, unrelenting, vulnerable
6.0	Classified, stylish, wet
6.5	Dashing, memorable, perfumed
7.0	Charismatic, creative, priceless

All seed words were within ${\pm }$ 0.1 from the Seed valence level.

#### Procedure

Participants read a letter of information and provided informed consent by clicking a ‘continue’ button. They then proceeded to the written completion task in which they were instructed to imagine a Group A and a Group B and were informed that they are a member of Group A. They were shown three seed words and were informed that this was how either their own group (Group A) or Group B described themselves. If the participants were informed that the seed words described Group A (their own group), then participants were asked to provide three more words for target Group A (the in-group condition). If participants were informed that the words described Group B, then participants were required to provide three more words for target Group B (the out-group condition). Participants typed their responses free-form in the provided text box, and submitted their responses by clicking the Submit button. The median completion time was 1.5 min. The task instructions below show the difference in instructions between conditions (in italics), while *[seed words]* represent one of the sets reported in Table 2.

Imagine two groups of people, Group A and Group B. In this task, you are part of Group A. Other people from *your group/group B* described themselves using the following words: *[seed words]*. Please add three more words below to describe *your own group./that group, group B*.

#### Independent variables

The critical independent variables were Target group (with in-group and out-group levels) and valence level.

#### Dependent variables

The dependent variable was the valence of the written responses to the completion task. These were estimated by associating words provided in each response with the 14,000-word database of valence ratings^53^. Since valence ratings were collected for lemmas (dictionary forms) only, to match them with the free forms provided by participants we assigned the same valence value to all inflected forms of a given word (e.g., the valence of the word *accept*, 6.59, was assigned to *accept, accepting, accepts*). This expanded the original database of valence ratings from roughly 14,000 word forms to 28,724 word forms.

#### Statistical power

Given that the reported median effect size in psychological research is Cohen’s $d =$ 0.4^67^, we aimed for a sample size of single-word responses that would provide a sufficiently high statistical power (*p* = 0.8) at the nominal 0.05 level of significance to allow detection of an effect size of *d* = 0.4 in a two-sample *t*-test for the global contrast between the two Target group conditions, i.e., in-group vs. out-group. A power calculation indicated that 99 responses per group (33 participants assuming that each participant provides three responses) was the required sample size for the comparison. While the study is overpowered for the purpose of detecting a statistically reliable global difference between responses for the in- and out-group, we acknowledge that the number of responses per each level of seed valence is slightly less than is required (see *n* per condition above) to offer a reliable estimate of statistical significance for the same group contrasts. However, we note that our interest is in the effect sizes of the contrasts at the individual seed levels, not in the statistical significance of the contrasts.

#### Data clean-up

We removed 76 typos, function words, and non-existing words from participant responses to the completion task (5.31% of all responses). This left a total of 1430 valid responses (an average of 79.44 responses per experimental condition). Of this pool of words, 1304 responses (91.19% of all valid responses) matched with words present in the database of valence ratings (603 word types in total).

### Experiment 2

A linear diffusion chain experiment was designed to examine the cumulative social evolution of intergroup bias expressed via linguistically encoded affective evaluations (see Fig. 1). Using the same task format as Experiment 1, we examined the transmission of intergroup bias down chains of multiple generations of in- and out-groups. The outcomes of interest were the changes in valence of words as they passed through the generations of participants. This experiment had a 2 (Group: in-group and out-group) × 3 (Valence: low, mid and high) × 5 (Generations) design. For each group (in vs. out), we initialized three chains which varied in their seed valence (low, mid, and high), i.e., Generation 1 received negative, neutral, or positive words as a description of a group to evaluate. In practice, we used output data from the low, mid, and high conditions from Experiment 1 as the first generation of responses. The first generation of responses were then used as the input (seed) for the second generation of responses, a process that was repeated for a total of five generations.

#### Participants

A total of 566 (*n* Generation 1 = 146; *n* Generation 2 = 115; *n* Generation 3 = 101; *n* = Generation 4 102; *n* = Generation 5 102) participants were recruited from Amazon’s Mechanical Turk crowdsourcing platform. As in Experiment 1, participants were randomly assigned to one of the two target group conditions (*n* in-group = 298; *n* out-group = 268). For each target group, participants were randomly assigned to a chain representing one of the three levels of seed valence (low, mid, high). Inclusion criteria and compensation were the same as in Experiment 1. Participants may have completed more than one task but only their first response was analyzed.

#### Stimulus materials

##### Generating the seed words (Generation 0)

We selected three levels of valence—the two extremes and the median (e.g., 3, 5, and 7)—and crossed them with the in-group and out-group manipulation. Experiment 1’s responses to seed valence levels 3, 5, 7 for the in-group and the out-group conditions were used as the stimuli for Generation 1.

##### Transmission of stimulus materials (Generations 1–5)

To form stimuli for Generation 2 in each of the six linear diffusion chains, we (i) considered all words produced in respective conditions of Generation 1 (i.e., Experiment 1), (ii) removed words in the bottom or top 5% of the distribution of valence values in each word set to minimize the influence of outliers, (iii) randomly selected 12 of remaining words, and (iv) after removing duplicates, provided the resulting set of 10–12 words as an input for Generation 2 (most of the resulting seed words were adjectives but there were also some nouns, e.g., *bigots*, *cheaters*, *leaders*). This algorithm was repeated after participants completed Generation 2 to form stimuli for Generation 3, and so on, until Generation 5 was complete. In this way, stimuli from Generations 2–5 were participant-generated.

Table 3 reports stimuli used in each chain: Stimuli shown to Generation 1 are the same as in respective conditions of Experiment 1.

**Table 3:** Seed words for Experiment 2 selected from the valence range ± 0.1 from Seed valence value.

Target generation	Target group	Low valence chain
1	In- and out-group	Despicable, guilty, slimy
2	In-group	compulsive, creepy, crooked, dignified, dishonest, hardworking, honorable, offensive, outcast, reprehensible, repulsive, shifty
2	Out-group	Creep, devious, dirty, disgusting, incompetent, lazy, nasty, offensive, shady, smelly, unsavory
3	In-group	Awful, calm, charismatic, cheaters, dedicated, different, generous, hardheaded, negative, outsider, overbearing, shady
3	Out-group	Crooked, dense, filthy, foul, horrible, idiotic, inept, mean, nasty, repugnant, sorry, ugly
4	In-group	Difficult, dramatic, empty, exacting, lazy, loyal, nerd, odd, patriotic, tough, unique
4	Out-group	Arrogant, bad, evil, impotent, nasty, pathetic, rough, sick, sloppy, stupid, unattractive, wicked
5	In-group	Boring, complicated, crazy, creative, detailed, intelligent, pretentious, quirky, smart, strange, trustworthy, weird
5	Out-group	Bigots, crude, disgusting, dumb, gross, lazy, sad, sadistic, ugly, unreliable
		Mid valence chain
1	In- and out-group	Acute, instantaneous, meticulous
2	In-group	Accurate, bright, cooperative, curious, detailed, diligent, exacting, focused, sharp, specific, speedy, spontaneous
2	Out-group	Analytical, careful, disruptive, focused, observant, picky, precise, quick, responsive, severe
3	In-group	Ambitious, attentive, caring, cheerful, efficient, friendly, funny, organized, researcher, resourceful, responsible, witty
3	Out-group	Active, agile, attentive, candid, compulsive, exacting, harsh, intense, particular, pretentious, strict
4	In-group	Daring, genuine, intelligent, inventive, logical, loyal, mindful, neat, optimistic, respectful, smart
4	Out-group	Aggressive, blunt, careless, dynamic, exact, observant, picky, resilient, rigid, strong, uncontrollable, vulgar
5	In-group	Capable, compassionate, creative, friendly, intuitive, traditional, real, reasonable, resilient, trustworthy, truthful
5	Out-group	Audacious, brutal, crazy, crude, fanatical, indecent, lewd, outspoken, overwhelming, scary, spontaneous, youthful
		High valence chain
1	In- and out-group	Charismatic, creative, priceless
2	In-group	Charming, compassionate, considerate, energetic, fair, humorous, innovative, kind, loyal, outgoing, powerful, thoughtful
2	Out-group	Charming, considerate, friendly, intelligent, leaders, outgoing, poised, reliable, selfless, talkative, thoughtful, valuable
3	In-group	Affectionate, calm, confident, creative, driven, humble, influential, intelligent, sensitive, smart, trustworthy
3	Out-group	Helpful, hospitable, ignorant, illiterate, intuitive, mindful, respectful, responsible, sensible, sincere, superstitious
4	In-group	Caring, cooperative, creative, efficient, friendly, genuine, hardworking, knowledgeable, open, outspoken, perceptive, respectable
4	Out-group	Accountable, considerate, dependable, dubious, friendly, funny, impartial, irrational, kind, passionate, retro, uneducated
5	In-group	Agreeable, cool, creative, humble, honorable, intelligent, kind, reliable, sincere, trustworthy, wise
5	Out-group	Adventurous, generous, humble, impressionable, juvenile, lazy, loyal, natural, nice, rebellious, spirited, spontaneous

#### Procedure

The task procedure was identical to Experiment 1, including the instructions. The only difference was that the input to Generation 1 consisted of three words initialized by researchers, while the inputs that were fed to Generations 2–5 were participant-generated and contained 10–12 words.

#### Independent variables

The list of independent variables included target group and valence level of Generation 1 (low = 3, mid = 5, or high = 7), as well as the ordinal number of Generation (1-5).

#### Dependent variables

The valence of the typed responses at each generation of each diffusion chain were measured using the lexical database described in the Methods section of Experiment 1.

### Data clean-up

We removed 78 typos, function words, and non-existing words from participant responses to the completion task (4.81% of all responses), leaving a total of 1620 valid responses (an average of 27 responses per experimental condition). Within this pool of words, 1503 responses (92.78% of all valid responses) matched with words present in the database of valence ratings (555 word types in total).

### Statistical considerations

To formally examine the effects of target group on the social transmission of verbal descriptions, we fitted a linear mixed-effects model with valence of the verbal descriptions as the outcome variable. The fixed effects included the interaction between generation (entered as a continuous predictor), target group (2 levels: in-group and out-group) and seed valence (entered as a categorical predictor with 3 levels: low, mid, and high). By-participant random intercepts were included as random effects. The mixed-effects regression model included restricted maximum likelihood (REML) estimations, which were computed using the lme4 package^68^ in R version 4.2.2^69^. Model *p*-values were obtained using the lmerTest package in R^70^ which uses Satterthwaite’s degrees of freedom method.

We began by fitting a simple model (with just main effects of each predictor) and then added in two-way interactions and the three-way interaction. Chi-square tests on the deviance statistics were used to test whether the interactions were justified. Additional parameters were included only if the more complex model showed a statistically significant improvement in model fit. The best fitting model included a significant three-way interaction between generation, seed valence and group. Further model criticism included refitting final models after removing outlying responses. This procedure involved refitting models while excluding absolute standardized residuals exceeding 2.5 standard deviations^71^.The emmeans package in R^72^ was used to decompose the interaction (all comparisons were adjusted with Tukey *p*-value adjustment method). Table 1 provides the omnibus effects of the final model which were calculated with Type III model comparisons using the car package^73^.

### Supplementary Information

Supplementary Tables.

## Data Availability

The datasets generated and analysed during the current study, along with R code used for statistical analysis, are available in the Open Science Framework repository, https://osf.io/argcu/.
