# Supplementary material for: A psycholinguistic study of intergroup bias and its cultural propagation
Source: Sci Rep. 2024 Apr 14;14:8613. doi: 10.1038/s41598-024-58905-y (PMC11016536; doi:10.1038/s41598-024-58905-y)
Supplement: Supplementary file 1 — Supplementary Tables. [file 41598_2024_58905_MOESM1_ESM.pdf]

Supplementary Materials

Table S1

Results of the linear mixed-effects regression model fitted to difference in valence from seed level in Experiment 1.

Fixed effect	$\hat{\beta}$	*SE*	*t*
Intercept	2.268***	0.286	7.941
Target group: out-group	-1.341**	0.394	-3.407
Generation	0.28***	0.055	-5.101
Seed valence: mid	0.152*	0.076	2.007

Random effect	*SD*
Participant	0.88

Conditional R^2	.484
Marginal R^2	.081
SD of the residual	0.99
N trials	1,304
N trials after trimming	1,286

Note. Target group dummy coded with in-group as the reference level. Seed valence dummy coded with low as the reference level.
$p < .05$. ** $p < .01$. *** $p < .001$.

Table S2

Results of the linear mixed-effects regression model fitted to response valence in Experiment 2.

Fixed effect	$\hat{\beta}$	*SE*	*t*
Intercept	3.598***	0.24	14.991
Target group: out-group	-0.424	0.335	-1.265
Generation	0.549***	0.075	7.318
Seed valence: mid	2.659***	0.323	8.223
Seed valence: high	3.367***	0.32	10.525
Target group: out-group x Generation	-0.616***	0.106	-5.796
Target group: out-group x Seed valence: mid	0.439	0.463	0.948
Target group: out-group x Seed valence: high	0.453	0.463	0.979
Generation x Seed valence: mid	-0.346**	0.1	-3.46
Generation x Seed valence: high	-0.512***	0.101	-5.056
Target group: out-group x Generation x Seed valence: mid	-0.011	0.146	-0.074
Target group: out-group x Generation x Seed valence: high	0.416**	0.148	2.81

Random effect	*SD*
Participant	0.849

Conditional R^2	.791
Marginal R^2	.596
SD of the residual	0.877
N trials	1,503
N trials after trimming	1,466

Note. Target group dummy coded with in-group as the reference level. Seed valence dummy coded with low as the reference level.
$p < .05$. ** $p < .01$. *** $p < .001$.
